# N-(4-methoxyphenyl) caffeamide-induced melanogenesis inhibition mechanisms

**DOI:** 10.1186/s12906-016-1554-6

**Published:** 2017-01-23

**Authors:** Yueh-Hsiung Kuo, Chien-Chia Chen, Po-Yuan Wu, Chin-Sheng Wu, Ping-Jyun Sung, Chien-Yih Lin, Hsiu-Mei Chiang

**Affiliations:** 1 Department of Chinese Pharmaceutical Sciences and Chinese Medicine Resources, China Medical University, Taichung, 404 Taiwan; 2 Department of Biotechnology, Asia University, Taichung, 413 Taiwan; 3 Department of Cosmeceutics, China Medical University, Taichung, 404 Taiwan; 4 Department of Dermatology, China Medical University Hospital, Taichung, 404 Taiwan; 5 School of Medicine, China Medical University, Taichung, 404 Taiwan; 6 National Museum of Marine Biology and Aquarium, Pingtung, 944 Taiwan

**Keywords:** *N*-(4-methoxyphenyl) caffeamide, Melanogenesis, Propolis, Microphthalmia-associated transcription factor, cAMP response element-binding protein, Glycogen synthase kinase 3 beta

## Abstract

**Background:**

The derivative of caffeamide exhibits antioxidant and antityrosinase activity. The activity and mechanism of *N*-(4-methoxyphenyl) caffeamide (K36E) on melanogenesis was investigated.

**Methods:**

B16F0 cells were treated with various concentrations of K36E; the melanin contents and related signal transduction were studied. Western blotting assay was applied to determine the protein expression, and spectrophotometry was performed to identify the tyrosinase activity and melanin content.

**Results:**

Our results indicated that K36E reduced α-melanocyte-stimulating hormone (α-MSH)-induced melanin content and tyrosinase activity in B16F0 cells. In addition, K36E inhibited the expression of phospho-cyclic adenosine monophosphate (cAMP)-response element-binding protein, microphthalmia-associated transcription factor (MITF), tyrosinase, and tyrosinase-related protein-1 (TRP-1). K36E activated the phosphorylation of protein kinase B (AKT) and glycogen synthase kinase 3 beta (GSK3β), leading to the inhibition of MITF transcription activity. K36E attenuated α-MSH induced cAMP pathways, contributing to hypopigmentation.

**Conclusions:**

K36E regulated melanin synthesis through reducing the expression of downstream proteins including *p*-CREB, *p*-AKT, *p*-GSK3β, tyrosinase, and TRP-1, and activated the transcription factor, MITF. K36E may have the potential to be developed as a skin whitening agent.

## Background

Melanin plays a pivotal role in preventing photodamage and photocarcinogenesis of the skin; however, abnormal accumulation of melanin induces hyperpigmentation disorders such as age spots and melasma [1, 2]. Melanogenesis is a series of complex process with many participating factors. Genetic background is the most crucial factor for skin pigmentation; more than 150 genes have been found to regulate melanin biosynthesis [3–5]. Moreover, nongenetic factors, such as medication, hormonal changes, inflammation, ageing, and exposure to ultraviolet (UV) irradiation, affect skin pigmentation [4, 5]. Melanogenesis is regulated by various proteins and enzymes including tyrosinase, microphthalmia-associated transcription factor (MITF), tyrosinase-related protein-1 (TRP-1), and tyrosinase-related protein-2 (TRP-2) [4, 6–8]. UV irradiation stimulates the secretion of α-melanocyte-stimulating hormone (α-MSH) in keratinocytes, which binds to the melanocortin 1 receptor (MC1R) and catalyses adenosine triphosphate conversion to cyclic adenosine monophosphate (cAMP) [9]. cAMP stimulates protein kinase A (PKA), and PKA translocates into the nucleus and activates cAMP-response element-binding protein (CREB) [10, 11]. Phospho-cAMP-response element binding protein (*p*-CREB) increases the expression of MITF to induce the expression of tyrosinase, TRP-1, and TRP-2. Tyrosinase undergoes maturation and activation through multiple mechanisms, including copper binding, glycosylation, and phosphorylation, resulting in melanin synthesis [12].

**Fig. 1:**
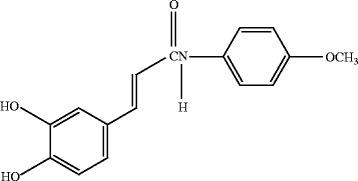
Structure of K36E

Substantial research has studied the regulation of melanin biosynthesis for the development of hypopigmenting agents. To inhibit tyrosinase activity and reduce melanin synthesis, several tyrosinase inhibitors that prevent hyperpigmenation have been developed through either synthesis or isolation from natural sources [1, 2, 7, 13]. *N*-(4-methoxyphenyl) caffeamide (K36E; Fig. 1) is an analogue of caffeic acid phenethyl ester, an active component of propolis. In our previous study, a caffeamide derivative exhibited antioxidant properties, prevented the degradation of skin collagen after UVB exposure, and stimulated collagen synthesis in human skin fibroblasts and in hairless mice [14, 15]. Another caffeamide derivative exhibited antimelanogenic activity by inhibiting tyrosinase activity and expression [16]. Moreover, caffeic acid derivatives such as *trans*-*N*-caffeoyltyramine and *trans*-*N*-dihydro-*p*-hydroxycinnamoyltyramine inhibited tyrosinase activity in melanocytes [17]. Thus, we speculated that K36E inhibits melanogenesis. In the current study, we investigated the effect of K36E activity on melanin synthesis in B16F0 cells, which is a well-established model for the investigation of skin whitening agents [18–20]. In addition, we investigated whether the antimelanogenic activity of K36E depends on the regulation of TRP-1, AKT/glycogen synthase kinase 3 beta (GSK3β)/CREB, and MITF.

## Methods

### Materials and chemicals

K36E was synthesized and identified by Professor Yueh-Hsiung Kuo with a purity of 99.9% [21]. α-MSH was purchased from Merck (Darmstadt, Germany). Arbutin, 3,4-dihydroxy-l-phenylalanine (L-DOPA), DL-dithiothreitol, H-89 dihydrochloride hydrate, phenylmethanesulfonyl fluoride, and L-tyrosine were purchased from Sigma-Aldrich Chemical Co. (St. Louis, MO, USA). Fetal bovine serum (FBS), Dulbecco’s modified Eagle’s medium (DMEM) and trypsin- ethylenediaminetetraacetic acid (EDTA) were purchased from the GIBCO Invitrogen Corporation (NY, USA). An antibody recognising MITF was obtained from Abcam (Cambridge, MA, USA). Antibodies recognising *p*-CREB and CREB were purchased from Cell Signalling Technology, Inc. (Danvers, MA, USA). Antibodies recognising phospho-AKT, AKT, and phospho-glycogen synthase kinase 3 beta (*p*-GSK3β) were obtained from GeneTex, Inc. (CA, USA). Antibodies recognising actin, GSK3β, TRP-1, and tyrosinase were obtained from Santa Cruz Biotechnology, Inc. (Santa Cruz, CA, USA).

### Effect of K36E on mushroom tyrosinase inhibition

The activity of mushroom tyrosinase was spectrophotometrically determined with minor modifications to the previously described procedure [7, 21–23]. Arbutin (2 mM) was the positive control. The test sample and L-tyrosine in phosphate buffer saline (PBS) and were added to a 96-well microplate (Nunc, Denmark), and mushroom tyrosinase was added. After incubation, the amount of dopachrome produced in the reaction mixture was determined at the optical density of 492 nm by using a microplate reader (Tecan, Grodig, Austria).

### Cell cultures

B16F0 cells were purchased from the Bioresource Collection and Research Centre in Taiwan and cultured in DMEM supplemented with 10% FBS and 100 units/mL of penicillin and streptomycin at 37 °C in 5% CO_2_.

### Cell viability assay

Cell growth experiments were performed using a 3-(4, 5-dimethylthiazol-2-yl)-2, 5-diphenyltetrazolium bromide (MTT) assay with minor modifications to the previously described procedure [7, 8, 24, 25]. Hydrogen peroxide was used as the positive control. The cells were cultured overnight and treated with various concentrations of K36E for 48 h, and an MTT solution was then added to each well. After incubation, a sodium dodecyl sulphate (SDS) solution was added, dissolving the formazan crystals produced in the cells. The optical density was measured at 570 nm by using a microplate reader (Tecan, Grodig, Austria).

### Cellular melanin content

The melanin content in B16F0 cells was measured by using a method modified from previous studies [7, 8, 23]. The B16F0 cells were seeded in 6-well plates at a density of 7 × 10^4^ cells per well and incubated overnight. The cells were exposed to a medium containing α-MSH and K36E for 48 h. Arbutin (1 mM) was the positive control. NaOH (2 N) was added to each well to lyse the cells, which were then centrifuged. The melanin content in the supernatant was measured at 405 nm by using an ELISA reader (Tecan, Grodig, Austria).

### Cellular tyrosinase activity assay

The tyrosinase activity of B16F0 cells after K36E treatment was measured with slight modification on the method described in previous studies [7, 26, 27]. B16F0 cells were plated in 24-well multidishes and incubated overnight. The cells were treated with various concentrations of K36E and were incubated for another 48 h. They were washed with PBS and lysed with 1% Triton X-100 mixed in 100 mM PBS (pH 6.8); the resultant mixture was frozen during incubation at −80 °C for 15 min and thawed at room temperature. Subsequently, the samples were centrifuged. A freshly prepared substrate (15 mM L-DOPA in a 48 mM pH 7.1 sodium phosphate buffer) was added to the supernatant and incubated. The absorbance was subsequently measured at 405 nm by using a microplate reader (Tecan, Grodig, Austria).

The rate of tyrosinase activity was calculated using the following equation:

$$ \mathrm{Activity}\left(\%\right)=\frac{{\mathrm{OD}}_{405\mathrm{sample}}}{{\mathrm{OD}}_{405\ \mathrm{control}}}\times 100 $$

### Western blot analysis

Western blot analysis was used to demonstrate the effects of K36E on the expression of melanogenesis-related proteins in B16F0 cells as previously described [7, 8, 22, 28, 29]. B16F0 cells were seeded in a 10-cm dish for 24 h and treated with α-MSH alone (control group) or with α-MSH plus various concentrations of K36E for 48 h. The lysates were centrifuged and the protein content was determined using a Bradford reagent (Bio-Rad, Hercules, CA, USA). Twenty micrograms of protein were separated on a sodium dodecyl sulfate polyacrylamide gel electrophoresis (SDS-PAGE) gel and blotted using a polyvinylidene difluoride (PVDF) membrane (Hybond ECL, Amersham Pharmacia Biotech Inc., Piscataway, NJ, USA). The blots were blocked with 5% (w/v) skimmed milk in Tris-buffered saline containing 0.05% Tween 20 and with specific antibodies: actin (1:1000), AKT (1:5000), *p*-AKT (1:5000), CREB (1:1000), *p*-CREB (1:1000), GSK3β (1:500), *p*-GSK3β (1:500), MITF (1:1000), TRP-1 (1:500), and tyrosinase (1:200). The PVDF membranes were incubated with the corresponding conjugated anti-immunoglobulin G horseradish peroxidase (Santa Cruz Biotechnology, Inc.). Immunoreactive proteins were detected using an Enhanced Chemiluminescence Plus kit (Fujifilm, LAS-4000), and signal strengths were quantified using a densitometric program (MultiGauge V2.2). The results of western blot assays represented at least three individual experiments.

### Statistical analyses

Values were expressed as the mean ± standard deviation from the results of at least three individual experiments. Differences in the effects of various treatments were compared using the Student’s *t*-test or ANOVA as well as Scheffe’s test through SPSS software (version 12.0). *P* values <0.05 indicated significance.

## Results

### Inhibition of mushroom tyrosinase activity by K36E

K36E at 1000 μM significantly reduced the mushroom tyrosinase activity. The mushroom tyrosinase inhibitory effect at 500, 750 and 1000 μM was 6.8% ± 1.6%, 14.0% ± 7.1% and 36.8% ± 1.1%, respectively. In addition, the inhibition rate of 2 mM arbutin on mushroom tyrosinase activity was 65.3% ± 2.5%.

### Effect of K36E on the viability of B16F0 cells

Cell viability after treatment with 1, 1.5, 2, 2.5, 4, 5, 10, 25, and 50 μM K36E was 92.7% ± 2.0%, 91.7% ± 2.1%, 90.9% ± 2.2%, 87.8% ± 4.2%, 72.8% ± 1.0%, 68.5% ± 2.4%, 54.6% ± 1.6%, 38.3% ± 0.8%, and 28.4% ± 2.3%, respectively. Hydrogen peroxide was the positive control, and the cell viability of 0.1 μM H_2_O_2_ was 48.9% ± 7.5% after 48 h treatment. The cell viability was acceptable for developing a material for cosmetics. According to International Organization for Standardization (ISO) 10993–5:2009 (Biological Evaluation of Medical Devices), cell viability higher than 80% is considered as noncytotoxicity. The results indicated that treatment with 0.5 to 2.5 μM K36E for 48 h had no cytotoxic effect on the B16F0 cells.

### Inhibition of melanin biosynthesis by K36E in B16F0 cells

**Fig. 2:**
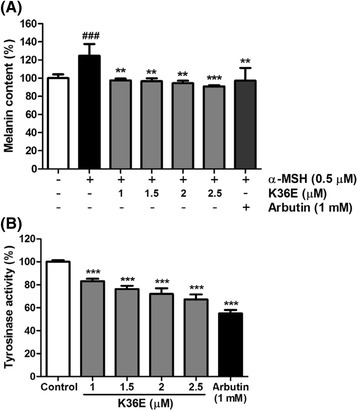
Effects of K36E on melanogenesis of B16F0 cells. **a** K36E reduced α-MSH-induced melanin content (%) of B16F0 cells. (Significant difference vs. control: ###, *P* < 0.001; significant difference vs. α-MSH-treated group: **, *P* < 0.01; ***, *P* < 0.001) and (**b**) tyrosinase activity (%) of B16F0 cells treated with K36E. (Significant difference vs. control: ***, *P* < 0.001)

Figure 2a shows the effects of K36E on melanin biosynthesis after stimulation by 0.5 μM α-MSH in B16F0 cells. The intracellular melanin content increased to 124.6% ± 13.0% after treatment with α-MSH. K36E at doses higher than 1.0 μM significantly reduced the melanin content, which decreased to 97.5% ± 1.9%, 96.6% ± 3.3%, 94.4% ± 2.8%, and 90.8% ± 1.4% (Fig. 2a). The effect of K36E on melanin biosynthesis was similar to that of 1 mM of arbutin.

### Inhibition of tyrosinase activity by K36E in B16F0 cells

K36E significantly inhibited tyrosinase activity in B16F0 cells after treatment for 48 h (Fig. 2b). The levels of tyrosinase activity were 83.2% ± 2.1%, 76.3% ± 2.9%, 72.0% ± 5.0%, and 67.2% ± 4.4% after treatment with 1, 1.5, 2, and 2.5 μM K36E, respectively, for 48 h. The results indicated that K36E inhibited the melanin content of B16F0 cells through the inhibition of tyrosinase activity.

### Effects of K36E on melanogenesis-related proteins

#### K36E downregulated tyrosinase and TRP-1 expression

**Fig. 3:**
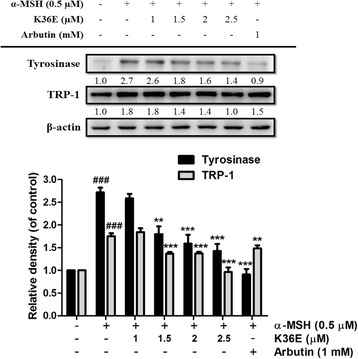
Effect of K36E on α-MSH-induced expression of tyrosinase and TRP-1 in B16F0 cells. (Significant difference vs. control: ###, *P* < 0.001; significant difference vs. α-MSH-treated group: **, *P* < 0.01; ***, *P* < 0.001)

To examine whether the inhibition of melanogenesis by K36E was related to the expression levels of melanogenesis-related proteins, including tyrosinase and TRP-1, B16F0 cells were incubated with α-MSH (0.5 μM) and various concentrations of K36E (1–2.5 μM) for 48 h. Although tyrosinase expression exhibited a 2.7-fold increase compared with that in the control after treatment with α-MSH, K36E significantly suppressed tyrosinase expression in a dose-dependent manner (Fig. 3). In addition, K36E significantly reduced α-MSH-stimulated TRP-1 expression at doses higher than 1.5 μM (Fig. 3).

#### K36E downregulated MITF expression

**Fig. 4:**
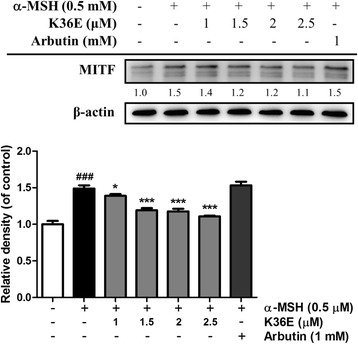
Effect of K36E on α-MSH-induced expression of MITF in B16F0 cells. (Significant difference vs. control: ###, *P* < 0.001; significant difference vs. α-MSH treated group: *, *P* < 0.05 ***, *P* < 0.001)

MITF expression in B16F0 cells exhibited a 1.5-fold increase compared with that in the control after treatment with α-MSH (Fig. 4). K36E treated for 4 h dose-dependently inhibited MITF expression and significantly downregulated MITF expression in the B16F0 cells at a concentration of 1 μM (Fig. 4).

#### K36E downregulated p-CREB expression

**Fig. 5:**
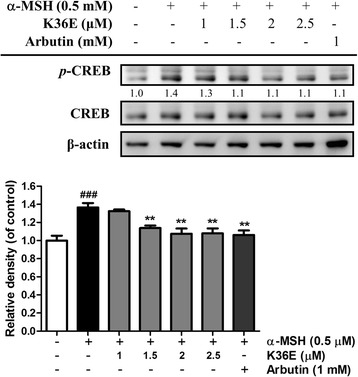
Effect of K36E on α-MSH-induced expression of *p*-CREB in B16F0 cells. (Significant difference vs. control: ###, *P* < 0.001; significant difference vs. α-MSH-treated group: **, *P* < 0.005)

*p*-CREB expression in B16F0 cells exhibited a 1.4-fold increase compared with that in the control after α-MSH treatment (Fig. 5). K36E significantly inhibited *p*-CREB expression at concentrations higher than 1.5 μM and subsequently downregulated MITF expression in the B16F0 cells.

### Effects of K36E on the melanogenesis signalling pathway

#### K36E-inhibited melanogenesis was associated with PKA regulation

**Fig. 6:**
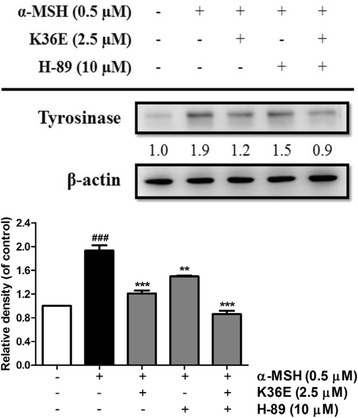
Effect of K36E on α-MSH-induced expression of tyrosinase in B16F0 cells after treatment with H-89. (Significant difference vs. control: ###, *P* < 0.001; significant difference vs. α-MSH-treated group: **, *P* < 0.01; ***, *P* < 0.001)

To determine whether K36E-inhibited melanogenesis was associated with PKA, B16F0 cells were incubated with 10 μM H-89, a PKA inhibitor [30], and 2.5 μM K36E for 48 h. Treatment with K36E and H-89 separately caused a 1.2- and 1.5-fold decrease in α-MSH-induced tyrosinase expression, respectively, compared with that in the control (Fig. 6). In addition, cotreatment with K36E and H-89 cause a 0.9-fold decrease in tyrosinase expression compared with that in the control. In addition, the tyrosinase expression following cotreatment was significant lower than that of K36E or H-89 treatment separately. The results indicated that the PKA pathway may be involved in the antimelanogenic effect of K36E.

#### K36E inhibited melanogenesis by upregulating p-AKT and p-GSK3β expression

**Fig. 7:**
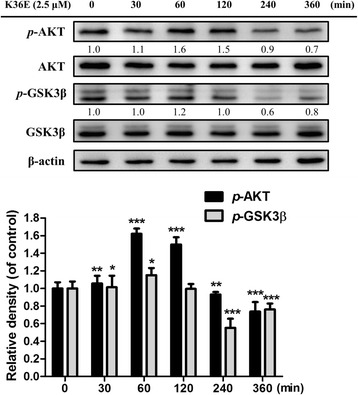
Effect of K36E on expression of p-AKT and p-GSK3β in B16F0 cells. (Significant difference vs. control: *, *P* < 0.05; **, *P* < 0.01; ***, *P* < 0.001)

As shown in Fig. 7, treatment with 2.5 μM K36E for 1 h markedly increased *p*-AKT and *p*-GSK3β expression. The level of *p*-AKT reached a maximum (1.6-fold increase compared with that in the control) after 1 h and 1.5-fold increase after 2 h; the level of *p*-GSK3β also displayed a 1.2-fold increase after 1 h compared with that in the control, suggesting that K36E suppressed melanogenesis in B16F0 cells by activating the AKT and GSK3β signalling pathways, resulting in the inhibition of MITF expression and transcription activity and, thus, inhibiting the expression of the tyrosinase gene.

## Discussion

Tyrosinase and its activity play a major role in controlling melanogenesis [31–33]. Agents or products that inhibit tyrosinase activity have been used in skin whitening cosmetics and cosmeceuticals [1, 2, 34]. Quercetin and vanillic acid inhibited α-MSH induced the expression of MITF, tyrosinase, TRP-1, and TRP-2, causing melanogenesis inhibition [35, 36]. Resveratrol derivatives inhibited melanin synthesis through the inhibition of melanogenic enzyme expressions such as tyrosinase and TRP-1 [37]. Our results indicated that K36E inhibited tyrosinase activity and α-MSH-induced protein expression, thereby suppressing melanin biosynthesis. In addition, K36E inhibited melanogenesis-related proteins such as TRP-1. TRP-1 is considered to play a vital role both as a structural protein and catalytic enzyme in the eumelanic pathway of melanosomes [38, 39]. The results mentioned above suggested that the decrease in melanogenesis of K36E could be achieved through its inhibition on the signalling pathway that regulates tyrosinase expression and activity.

**Fig. 8:**
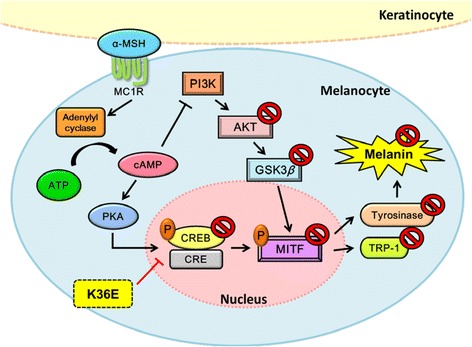
Schematic diagram showing inhibitory effects of K36E in α-MSH-induced melanogenesis

MITF is the most critical transcription factor that regulates melanogenesis by inducing the expression of melanogenic genes [5, 40]. MITF activation upregulates the expression of tyrosinase and TRP-1, and it consequently increases melanin synthesis. In this study, K36E suppressed melanogenesis by inhibiting α-MSH induced MITF expression. The cAMP pathway plays a pivotal role in α-MSH-induced melanogenesis. In previous study, cAMP-elevating agents inhibited PI3K/AKT. GSK3β may stimulate the binding of MITF to its target sequence to stimulate the expression of melanogenic enzymes and facilitate melanin production [41]. cAMP inhibits PI3K and AKT phosphorylation and activity, and reduces GSK3β phosphorylation to stimulate its activity. The activation of cAMP signal transduction results in the binding of MITF to the tyrosinase promoter, thereby leading to the stimulation of melanogenesis [41, 42]. Activation of the AKT pathway suppressed melanin synthesis by decreasing melanogenic enzymes [41]. Cordycepin was reported to inhibit α-MSH and IBMX induced melanin biosynthesis by inhibiting melanin synthesis related enzymes, such as tyrosinase, TRP-1, and TRP-2, suppressing CREB and MITF activity, and activating the PI3K/AKT pathway in B16F10 melanoma cells [43]. In our results, K36E inhibited CREB phosphorylation. K36E elevated the expression of *p*-AKT and *p*-GSK3β, possibly reducing MITF transcription to suppress tyrosinase gene expression. Previous studies have reported that the activation of AKT inhibited melanogenesis in melanocytes [33, 44]. Thus, K36E inhibited α-MSH induced hyperpigmentation caused by AKT and GSK3β activation, and subsequently downregulated MITF, CREB, tyrosinase, and TRP-1 production (Fig. 8).

UV exposure stimulates the secretion of α-MSH in keratinocytes. α-MSH binds to MC1R in melanocytes, resulting in cAMP production and PKA activation [10]. The signal transduction related to the cAMP pathway, including the activation of PKA and CREB transcription factors, leads to the upregulation of MITF [45]. PKA subsequently phosphorylates CREB to activate MITF gene expression [46, 47]. Nicotinic acid hydroxamate inhibited melanin synthesis through the activation of the MEK/ERK and AKT/GSK3β signalling pathways in B16F10 melanoma cells [48]. Dried pomegranate concentration powder exerts whitening effects by effectively decreasing tyrosinase activity and melanin production in B16F10 cells through inactivation of the p38 and PKA/CREB signalling pathways in B16F10 cells [49]. cAMP-induced PI3K inhibition decreases AKT phosphorylation and its activation. In the present study, α-MSH-induced MITF expression was inhibited by K36E and H-89, which is a PKA inhibitor. In addition, cotreatment with K36E and H-89 significantly attenuated the K36E-induced reduction of melanin synthesis. Our results suggested that the antimelanogenic activity of K36E is associated with PKA pathway and thus leads to downregulation of MITF (Fig. 8).

## Conclusion

K36E reduced MITF expression by inhibiting CREB phosphorylation. Additionally, K36E inhibited MITF expression by upregulating the phosphorylation of AKT and GSK3β, which subsequently inhibited the expression of tyrosinase and TRP-1 and thereby reduced melanin biosynthesis. Normal melanocytes and in vivo studies may be applied for further investigation into the effect of K36E on melanogenesis. In conclusion, K36E may be a candidate for the regulation of melanogenesis and it is likely to have various applications in skin whitening products in the future.
